# Distinguishing HIV-1 drug resistance, accessory, and viral fitness mutations using conditional selection pressure analysis of treated versus untreated patient samples

**DOI:** 10.1186/1745-6150-1-14

**Published:** 2006-05-31

**Authors:** Lamei Chen, Christopher Lee

**Affiliations:** 1 Institute for Genomics & Proteomics, Molecular Biology Institute, Dept. of Chemistry & Biochemistry, UCLA, Los Angeles, CA 90095-1570, USA

## Abstract

**Background:**

HIV can evolve drug resistance rapidly in response to new drug treatments, often through a combination of multiple mutations [1-3]. It would be useful to develop automated analyses of HIV sequence polymorphism that are able to predict drug resistance mutations, and to distinguish different types of functional roles among such mutations, for example, those that directly cause drug resistance, versus those that play an accessory role. Detecting functional interactions between mutations is essential for this classification. We have adapted a well-known measure of evolutionary selection pressure (*K*_*a*_/*K*_*s*_) and developed a conditional *K*_*a*_/*K*_*s *_approach to detect important interactions.

**Results:**

We have applied this analysis to four independent HIV protease sequencing datasets: 50,000 clinical samples sequenced by Specialty Laboratories, Inc.; 1800 samples from patients treated with protease inhibitors; 2600 samples from untreated patients; 400 samples from untreated African patients. We have identified 428 mutation interactions in Specialty dataset with statistical significance and we were able to distinguish primary vs. accessory mutations for many well-studied examples. Amino acid interactions identified by conditional *K*_*a*_/*K*_*s *_matched 80 of 92 pair wise interactions found by a completely independent study of HIV protease (p-value for this match is significant: 10^-70^). Furthermore, *K*_*a*_/*K*_*s *_selection pressure results were highly reproducible among these independent datasets, both qualitatively and quantitatively, suggesting that they are detecting real drug-resistance and viral fitness mutations in the wild HIV-1 population.

**Conclusion:**

Conditional *K*_*a*_/*K*_*s *_analysis can detect mutation interactions and distinguish primary vs. accessory mutations in HIV-1. *K*_*a*_/*K*_*s *_analysis of treated vs. untreated patient data can distinguish drug-resistance vs. viral fitness mutations. Verification of these results would require longitudinal studies. The result provides a valuable resource for AIDS research and will be available for open access upon publication at

**Reviewers:**

This article was reviewed by Wen-Hsiung Li (nominated by Eugene V. Koonin), Robert Shafer (nominated by Eugene V. Koonin), and Shamil Sunyaev.

## Open peer review

Reviewed by Wen-Hsiung Li (nominated by Eugene V. Koonin), Robert Shafer (nominated by Eugene V. Koonin), and Shamil Sunyaev. For the full reviews, please go to the Reviewers’ comments section.

## Background

During the past two decades, researchers and clinicians have made enormous efforts to identify drug resistance mutations in HIV-1 protease, a major target of anti-retroviral therapy. Discovery of a new drug resistance mutation typically requires a combination of clinical research (e.g. AIDS patients displaying drug resistance), molecular biology (e.g. sequencing), and biochemistry (e.g. obtaining viral samples and performing phenotypic assays). Over the last 20 years, many mutations and combinations of mutations in protease have been reported to play a role in drug resistance [4,5]. But this discovery process is laborious, expensive, and far from automatic – indeed, it is *research*. Meanwhile, HIV is constantly evolving new drug resistance mutations, often within weeks of introduction of a new drug [6-8]. Thus, a completely automated approach for identifying drug resistance mutations would be valuable [9,10].

Differences in reproductive success (such as the enhanced survival of a drug-resistant mutant) are reflected in *selection pressure*, which can be measured directly from sequence data. For example, *K*_*a*_/*K*_*s *_is a well-known metric of selection pressure for amino acid mutations, measured relative to the observed frequency of synonymous mutations (which cause no change to protein sequence or function) [11,12]. Ordinarily *K*_*a*_/*K*_*s *_values much less than one are observed, indicating that most amino acid mutations are deleterious. This is referred to as "negative selection". By contrast, *K*_*a*_/*K*_*s *_values greater than one indicate that amino acid mutations *improve *reproductive success (positive selection), a very unusual condition. By screening for individual amino acid mutations in HIV protease that displayed positive selection, this metric was able to correctly predict 19 out of 23 known drug resistance mutation sites in HIV protease [10]. This approach has several advantages: it is completely automatic; it is based on a large and well-understood body of evolutionary theory; it only requires raw sequence data to perform this analysis.

However, not all positively selected mutations are created equal. It would be very useful to be able to distinguish different types of mutations on the basis of their interactions with other sites. For example, a binding site mutation that interacts directly with the drug molecule might confer primary drug resistance [13-15]. Alternatively, a mutation that interacts with another site within HIV protease might compensate for destabilizing effects of mutations at the other site [16,17]. Unfortunately, selection pressure metrics such as *K*_*a*_/*K*_*s *_provide no explicit mechanism for taking such interactions into account.

To address this problem, we have extended the *K*_*a*_/*K*_*s *_concept to consider interactions with other sites, by formulating a *conditional K*_*a*_/*K*_*s *_that measures the effects of mutations at one site on the selection pressure for mutations at another site. We have applied it to four completely independent datasets: **Specialty**: 50,126 HIV-1 positive patient plasma samples sequenced by Specialty Laboratories, Inc. between October 1999 and October 2003 [10]. This dataset represents a mix of treated and untreated patient samples from the U.S. **Treated**: 1797 samples collected by Shafer and colleagues, from U.S. patients undergoing specific drug treatments [9]. **Untreated**: 2628 samples collected by Shafer and colleagues specifically from untreated patients [9]. **Africa**: 399 African HIV-1 subtype C samples downloaded from Los Alamos HIV Sequence Database [18-20]. These samples predominantly represent untreated patients. These sequences cover the whole protease region (codons 1 – 99) of HIV-1. The conditional Ka/Ks results are highly reproducible among these independent datasets. They reveal complex interactions between amino acid sites, and distinguish different types of mutations such as primary drug-resistance mutations vs. accessory mutations.

## Methods

### HIV-1 sequence data

We filtered all four datasets to exclude contamination by other HIV-1 subtypes. We compared all of the sequences in these datasets against HIV-1 subtype reference sequences from the Los Alamos database by using the program BLAST and assigned each sample to the subtype with the highest identify. All non-subtype B sequences were removed from the U.S. datasets and all non-subtype C sequences were removed from the African dataset. There might be multiple samples from the same individual in Specialty dataset. To eliminate the phylogenetic relationships among sequences, we also excluded samples that have 98% or greater nucleotide sequence similarity to each other.

Multiple sequence alignment and mutation detection were performed as previously described [10]. We used the widely accepted nucleotide reference sequence from the Los Alamos HIV Sequence Database as the "wildtype" for HIV-1 subtype B [21,22] and used the nucleotide consensus of all the African subtype C samples as the "wildtype" for HIV-1 subtype C. In this paper, we considered only single nucleotide substitutions; all other mutations were excluded.

### Unconditional Ka/Ks measurement for individual codon positions

Amino acid selection pressure can be calculated for an individual codon [23,24]; we will refer to this as the "unconditional *K*_*a*_/*K*_*s*_" to distinguish it from the conditional approach presented in the next section. We first measured the transition and transversion frequencies *f*_*t *_and *f*_*v *_from the entire dataset, according to



where *S *is the total number of samples, *N*_*t *_and *N*_*v *_are the number of observed transition and transversion mutations respectively, *n*_*t *_is the number of possible transitions in the region that was sequenced (simply equal to its length *L *in nucleotides), and *n*_*v *_is the number of possible transversions (equal to 2*L*). In this calculation (and all others below) we only counted single nucleotide substitutions; all other mutations were excluded. We performed the calculation of unconditional *K*_*a*_/*K*_*s *_as previously described [10], as the ratio of *N*_*a*_, the count of samples with amino acid mutations observed at that codon, over *N*_*s*_, the count of samples with synonymous mutations observed at that codon. This *N*_*a*_/*N*_*s *_ratio is then normalized by the ratio expected under a random mutation model (i.e., in the absence of any selection pressure), according to the following formula:



where *n*_*a*,*t *_is the number of possible transition mutations in the codon that would change the wildtype amino acid, *n*_*s*,*t *_is the number of possible transition mutations in the codon that are synonymous, and *n*_*a*,*v *_and *n*_*s*,*v *_are the equivalent numbers for transversions. When the observed ratio (*N*_*a*_/*N*_*s*_) is greater than the expected ratio (in the denominator of the expression above), the selection pressure *K*_*a*_/*K*_*s *_is greater than 1 and we say that the codon is under positive selection pressure.

We calculated an LOD confidence score for a codon to be under positive selection pressure according to the following formula:



where *N *is the total number of mutations observed in the codon, and *q *is calculated as follows:



### Conditional selection pressure analysis

To measure how mutation at one site alters the selection pressure at another site, we define the "conditional selection pressure" for a site *Y *in the presence of an amino acid mutation at another site *X *as:



where *N*_*YaXa *_is the number of samples with an amino acid mutation at codon *Y *and an amino acid mutation on codon *X*; and *N*_*YsXa *_is the number of samples with a synonymous mutation at codon *Y *and an amino acid mutation on codon *X*. We will refer to (*K*_*a*_/*K*_*s*_)_*Y*|*Xa *_as the "conditional *K*_*a*_/*K*_*s*_" at *Y *given an amino acid mutation at *X*.

We define the "conditional selection ratio" as the ratio of the conditional Ka/Ks divided by the selection pressure at *Y *measured in the absence of any mutation at *X*:



where *N*_*YaXo *_and *N*_*YsXo *_are the numbers of samples containing either an amino acid mutation or synonymous mutation at *Y *and no mutation at codon *X*.

### Confidence scoring for conditional selection pressure analysis

We screened for codon pairs with conditional Ka/Ks and conditional selection ratios above 1. To evaluate the statistical significance of apparent positive conditional selection pressure, we defined a LOD score based on the likelihood of observing at least *N*_*YaXa *_samples with amino acid mutations at codon *Y *and *X*, assuming neutral selection at codon *Y*:



where *N *= *N*_*YaXa *_+ *N*_*YsXa*_; and *q *as defined above.

To evaluate the statistical significance of the conditional selection ratio, we used a one-sided Fisher's exact test to measure the p-value for the null hypothesis that (*K*_*a*_/*K*_*s*_)_*Y*|*Xa *_≤ (*K*_*a*_/*K*_*s*_)_*Y*|*Xo*_. We performed Fisher's exact test on a 2-by-2 contingency table containing the counts *N*_*YaXa*_-1, *N*_*YsXa*_, *N*_*YaXo *_and *N*_*YsXo*_, using the statistical software package R. We deducted a single count from *N*_*YaXa *_to make the test stricter against cases with small numbers of counts.

Throughout this study, we used the following criteria for high confidence positive conditional selection pressure: (*K*_*a*_/*K*_*s*_)_*Y*|*X *_> 1, (*K*_*a*_/*K*_*s*_)_*Y*|*Xa *_> 1, *LOD *> 3, and *p *< 10^-3^.

### Comparing *K*_a_*/K*_*s*_ values between datasets

To test whether the difference in *K*_*a*_/*K*_*s *_value computed from two datasets *D *and *D' *was statistically significant, we calculated a p-value using a one-sided Fisher's exact test, using the 2 × 2 contingency table representing the counts of amino acid mutations and synonymous mutations from the two datasets: for unconditional Ka/Ks, (*N*_*a*_, *N*_*s*_, *N'_a_, N'_s_*); for conditional *K*_*a*_/*K*_*s*_, (*N*_*YaXa*_, *N*_*YsXa*_, *N'*_*YaXa*_, *N'*_*YsXa*_). We used a p-value threshold of 0.01.

## Results

### Detection of selection pressure interactions via conditional *K*_*a*_/*K*_*s*_

To dissect the functional roles of amino acid mutations in HIV protease, we analyzed the Specialty dataset of 48,387 HIV-positive samples for conditional *K_a_/K_s_* relationships. We screened all possible codon pairs using several criteria: positive conditional selection pressure (with a statistical significance test of LOD >3), and a conditional selection ratio greater than 1 (with a statistical significance test of p < 10^-3^). 400 codon pairs showed strong conditional relationships by these criteria (Table 1). These effects ranged from relatively mild increases (for example, the presence of an amino acid mutation at position 88 increased the *K*_*a*_/*K*_*s *_value at codon 10 by about 27%) to very large (at position 30, the *K*_*a*_/*K*_*s *_value increased from 0.4 to 71.1 depending on the absence or presence of an amino acid mutation at codon 88).

**Table 1:** Positive conditional selection pressure in HIV-1 protease

Codon Y	Codon X	N_XaYa_	N_XaYs_	(*K*_*a*_/*K*_*s*_)_Y|Xa_	N_XoYa_	N_XoYs_	(*K*_*a*_/*K*_*s*_)_Y|Xo_	(*K*_*a*_/*K*_*s*_)_Y|X_	LOD	p
90	73	1879	6	367.63	6842	628	12.79	28.74	>300	<0.001
82	24	564	0	277.79	3625	680	2.63	105.8	98.09	<0.001
90	95	161	1	189	7757	595	15.3	12.35	52.35	<0.001
82	48	483	2	118.95	3727	663	2.77	42.96	79.90	<0.001
82	53	366	2	90.13	4005	694	2.84	31.71	59.79	<0.001
82	55	332	2	81.76	3994	701	2.81	29.14	53.96	<0.001
30	88	1730	9	71.1	727	677	0.4	179	217.97	<0.001
63	50	132	1	65.01	22576	2037	5.46	11.91	21.31	<0.001
90	89	329	6	64.37	7677	524	17.2	3.74	100.28	<0.001
71	11	126	0	62.06	8775	964	4.48	13.84	21.91	<0.001
90	92	315	6	61.63	7706	575	15.73	3.92	95.67	<0.001
63	73	1711	14	60.2	20832	2016	5.09	11.83	269.97	<0.001
33	83	49	1	57.52	2436	803	3.56	16.15	15.09	<0.001
90	84	2039	45	53.19	6756	596	13.31	4	>300	<0.001
90	85	395	9	51.52	8361	627	15.65	3.29	117.75	<0.001

Due to the long list of positive conditional selection pressure detected in the Specialty dataset, only the 15 positive selections with the strongest increase in (*K*_*a*_/*K*_*s*_)_Y|Xa _value after conditioning were showed here. Please see the Additional File for the complete list.

Codon X: the codon site where the first mutation appears.

Codon Y: the codon site where the second mutation appears.

(*K*_*a*_/*K*_*s*_)_Y|Xa_: conditional selection pressure of codon Y in the presence of an amino acid mutation at codon X.

(*K*_*a*_/*K*_*s*_)_Y|Xo_: conditional selection pressure of codon Y in the absence of any mutation at codon X.

LOD: confidence score for (*K*_*a*_/*K*_*s*_)_Y|Xa _> 1.

p: p-value for (*K*_*a*_/*K*_*s*_)_Y|X _> 1 to be random.

**Figure 1:**
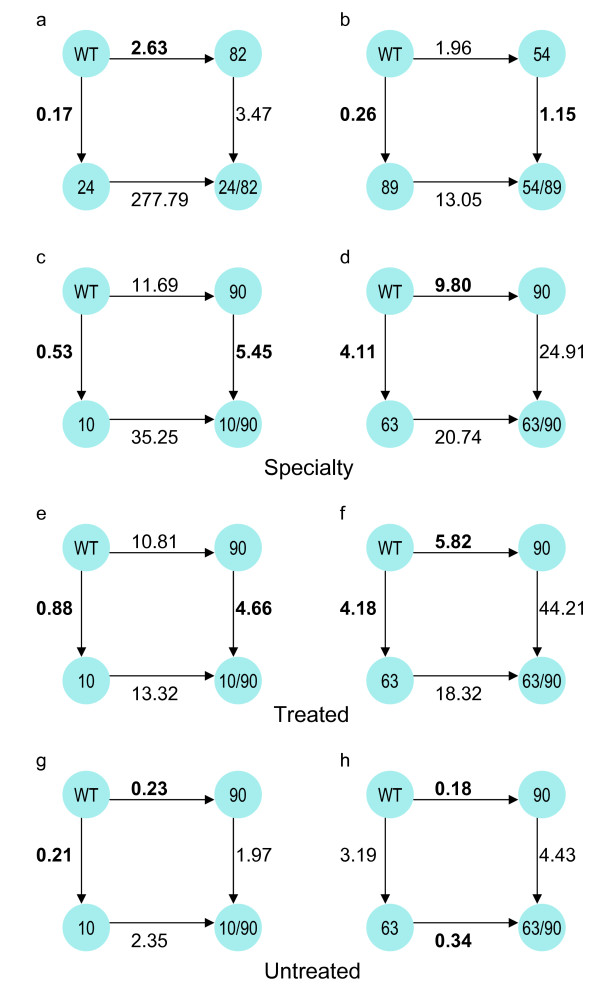
**Conditional Ka/Ks measurements for primary vs. accessory drug-resistance mutations**. For the two possible pathways from wildtype to a double mutant, we computed the conditional *K*_*a*_/*K*_*s *_values for each mutation conditioned on the presence or absence of the other mutation (shown as numbers next to each edge in the figure). Primary drug resistance codons are highlighted in bold. **a**) 24/82; **b**) 54/89; **c, e, g**) 10/90; **d, f, h**) 63/90. **a-d **are from the Specialty dataset; **e-f **from the Treated dataset; and **g-h **from the Untreated dataset (see text).

These data reveal a basic distinction between two types of positive selection effects: those that depend on the presence of another amino acid mutation at a specific site, versus those that do not. For example, amino acid mutations at codon 90 are strongly selected for, regardless of the presence or absence of a mutation at codon 10 (Fig. 1). By contrast, codon 10 displayed negative selection in samples with no mutation at 90, but strongly positive selection in samples containing an amino acid mutation at 90. These data show that mutations at 90 are directly selected by drug treatment, that mutations at 10 are not favorable by themselves, but that they become advantageous in viruses bearing a 90 mutation. These results closely match previous experimental studies showing that mutations at 90 cause drug resistance, while mutations at 10 have an accessory effect of compensating for the destabilizing effect of mutations at 90 [25]. We have observed similar asymmetric effects at other sites, for example 24/82 (Fig. 1a). In general, primary drug resistance mutations showed consistent positive selection, whereas accessory mutations often showed only conditional positive selection, dependent on the presence of a primary drug resistance mutation. By exposing such asymmetries, the conditional *K*_*a*_/*K*_*s *_analysis may be able to predict whether mutations have a primary drug resistance vs. accessory effect. For example, mutations at position 54 are positively selected, which in turn induce positive selection for mutations at 89, which by themselves are unfavorable (Fig. 1b). These data suggest that 89 is an accessory position, whose mutations can stabilize mutations at 54 (which are known to cause drug resistance [26]).

### Comparison with independent drug treatment studies of HIV protease

One weakness of the Specialty dataset is that it includes no information about individual drug treatments. To assess the significance of our conditional *K*_*a*_/*K*_*s *_results, we have compared them with the independent experimental study of Wu et al., who identified correlated mutation pairs, i.e. pairs of codons where mutations co-occurred much more frequently in patients with specific drug treatments, than in patients who received no drug treatment [9]. By comparing 1,004 HIV isolates from untreated patients with 1,240 patients from patients treated with one or more protease inhibitors, Wu et al. identified 92 protease codon pairs with significant correlated mutations associated with drug treatment. Our conditional *K*_*a*_/*K*_*s *_analysis of the Specialty dataset matched these results closely, identifying 80 of these 92 codon pairs as having positive conditional selection pressure (LOD>2 and p < 0.01). This result had strong statistical significance (p-value = 10^-70^). Thus conditional *K*_*a*_/*K*_*s *_appears to robustly detect mutational interactions that are genuinely associated with drug treatment, even from a dataset (Specialty) lacking any drug treatment information.

### Distinguishing drug-resistance vs. fitness mutations by comparison of *K*_*a*_/*K*_*s *_for treated vs. untreated datasets

We have sought to distinguish mutations that are selected specifically in response to drug treatment, from fitness mutations that are positively selected even in the absence of drug treatment [27]. To do so, we first compared unconditional *K*_*a*_/*K*_*s *_values from the Treated dataset with those from the Untreated dataset (Table 2). Nine codons underwent a confident shift from negative selection in Untreated, to positive selection in Treated. Of these, five (30, 53, 54, 82, and 90) are known primary drug-resistance mutations, and three (10, 24, 73) are known accessory drug-resistance mutation codons [28]. Thus, this shift appears to be an accurate predictor of sites involved in drug-resistance. Intriguingly, the one remaining codon (74) is not currently known to cause drug-resistance, but showed confident positive selection both in the Treated (*K*_*a*_/*K*_*s *_= 3.75) and Specialty (*K*_*a*_/*K*_*s *_= 5.87) datasets.

**Table 2:** Significant differences of selection pressure between Treated and Untreated dataset

Position	Treated				Untreated				p-value
		
	*K*_*a*_/*K*_*s*_	N_a_	N_s_	LOD	*K*_*a*_/*K*_*s*_	N_a_	N_s_	LOD	
***10*^*a*^**	**1.47**	**516**	**176**	**5.4**	**0.21**	**172**	**401**	**71.57**	**< 0.0001**
11	0.17	15	43	10.38	0.04	7	90	35.47	0.0017
17	0.06	17	142	49.46	0.16	55	176	40.54	0.0006
19	2.9	183	97	17.72	1.72	278	248	9.45	0.0004
*20*	0.39	137	131	12.56	0.07	41	215	80.54	< 0.0001
23	0.25	27	163	14.25	0.08	11	222	35.57	0.0006
***24***	**1.59**	**85**	**64**	**2.49**	**0.33**	**22**	**79**	**6.73**	**< 0.0001**
***30***	**1.84**	**211**	**43**	**4.1**	**0.18**	**22**	**46**	**12.11**	**< 0.0001**
*32*	0.53	49	46	3.08	0.01	2	69	31.72	< 0.0001
*48*	0.71	72	51	1.62	0.02	3	82	36.4	< 0.0001
*50*	0.9	27	13	0.51	0.07	2	12	5.74	0.0007
***53***	**2.99**	**48**	**6**	**2.44**	**0.31**	**5**	**6**	**1.91**	**0.0032**
***54***	**1.55**	**246**	**69**	**3.25**	**0.03**	**7**	**112**	**50.03**	**< 0.0001**
58	0.1	46	164	53.2	0.01	3	207	113.48	< 0.0001
61	0.1	55	211	70.15	0.05	63	431	172.9	0.0031
62	13.65	419	2	8.93	2.42	446	12	3.38	0.0094
*63*	6.1	1111	91	100.36	3.23	1343	208	71.62	< 0.0001
66	0.01	12	385	181.09	0.00	1	551	286.44	0.0002
67	0.2	64	118	26.07	0.10	75	290	95.99	0.0002
*71*	6.99	601	43	58.9	1.11	195	88	0.63	< 0.0001
***73***	**1.81**	**130**	**36**	**3.17**	**0.05**	**5**	**48**	**18.6**	**< 0.0001**
**74**	**3.75**	**75**	**10**	**5.36**	**0.22**	**7**	**16**	**4.09**	**< 0.0001**
76	1.02	34	40	0.28	0.16	10	74	11.25	< 0.0001
*77*	24.42	635	13	91.3	9.72	700	36	79.13	0.0025
***82***	**4.58**	**339**	**37**	**25.9**	**0.46**	**51**	**56**	**4.77**	**< 0.0001**
83	0.15	18	45	13.27	0.03	10	107	49.35	0.0006
*84*	1.46	112	5	0.56	0.00	0	14	300	< 0.0001
85	1.39	48	15	0.79	0.11	3	12	5.01	0.0001
*88*	0.82	154	70	1.11	0.1	25	95	32.49	< 0.0001
***90***	**12.26**	**472**	**46**	**107.02**	**0.25**	**12**	**58**	**6.9**	**< 0.0001**
92	0.1	34	131	44.36	0.04	25	211	90.47	0.0044

^a ^Mutations at codon in Italic are known associated with drug resistance. The nine codons that underwent a confident shift from positive selection in Treated to negative selection in Untreated are in bold.

A total of fifteen codons were positively selected in both Treated and Untreated dataset (Table 3; codon 71 has *K*_*a*_/*K*_*s *_value of 1.11 but a LOD = 0.63 in the Untreated dataset). The fact that these mutations were positively selected even in the absence of drug treatment suggests that they contribute directly to viral fitness. Of these, five (19, 62, 63, 71 and 77) displayed a statistically significant increase in *K*_*a*_/*K*_*s *_from Untreated to Treated (Table 2). Codons 63, 71 and 77 are known accessory drug-resistance mutations [28]. The functions of the remaining two codons are unknown. The significant increase of *K*_*a*_/*K*_*s *_value in the Treated dataset predicts an accessory role in drug-resistance.

**Table 3:** Positive selection pressure in Treated and Untreated datasets

	Selection pressure			Conditional selection pressure		
Treated	+^a^	+	-	+^a^	+	Not + selected, or not present
Untreated	+	-^b^	+	+	Not + selected, or not present	+
Codon	12,13,19,33,35,37,41,57,60,62,63,6477,93 71^c^	10,24,30,54,73,74,82,90 53^d^	None	19, 63, 71 93^e^	10, 24, 30, 35, 48, 54, 73, 82, 88, 90	None

^a ^positively selected. For selection pressure, *K*_*a*_/*K*_*s *_> 1 and LOD > 2; for conditional selection pressure, (*K*_*a*_/*K*_*s*_)_Y|Xa _> 1, (*K*_*a*_/*K*_*s*_)_Y|X _> 1, LOD > 3 and p < 0.001.

^b ^negatively selected (*K*_*a*_/*K*_*s *_< 1 and LOD > 2)

^c ^codon 71 is positively selected in Treated samples (*K*_*a*_/*K*_*s *_> 1 and LOD > 2), and it has a *K*_*a*_/*K*_*s *_value greater than 1 but a LOD score less than 2 in Untreated samples.

^d ^codon 53 is positively selected in Treated samples (*K*_*a*_/*K*_*s *_> 1 and LOD > 2), and it has a *K*_*a*_/*K*_*s *_value less than 1 but a LOD score less than 2 in Untreated samples.

^e ^In Treated dataset, codon 93 is positively selected with (*K*_*a*_/*K*_*s*_)_Y|Xa _> 1, (*K*_*a*_/*K*_*s*_)_Y|X _> 1, LOD > 3 but p > 0.001.

Strikingly, all of the known primary drug resistance mutations were observed to be negatively selected in the Untreated dataset, suggesting that they reduce fitness in the absence of drug treatment [29-31]. These negative selection effects were strongest for mutations at codons 66 (*K*_*a*_/*K*_*s *_< 0.01), 84(< 0.01), 32 (0.01), 48 (0.02) and 54 (0.03), and weakest at codons 82 (*K*_*a*_/*K*_*s *_= 0.46), 53 (0.31), 24(0.33), and 90 (0.25). Thus, in the absence of continuing drug treatment, these mutations would be expected to be gradually lost from the evolving viral population due to their greatly reduced fitness [29-31]. By contrast, while most accessory drug-resistance mutations were negatively selected in Untreated, some were neutral (e.g. 71), while others were strongly positively selected (e.g. 63, 77) even in the absence of drug treatment. This may explain why mutations at position 63 appear to be becoming dominant in the HIV population [26,32]

It is interesting to note that at no codon did we ever observe the pattern of positive selection in Untreated but negative selection in Treated (Table 3). Thus, drug-treatment appears to preserve positive selection pressure for fitness mutations that are found in the untreated dataset, merely adding new positive selection effects for drug-resistance.

Although the Treated and Untreated datasets are much smaller than Specialty, we were able to calculate conditional *K*_*a*_/*K*_*s *_values for many sites. Fig. 1e–h shows a comparison of conditional *K*_*a*_/*K*_*s *_results from the Treated and Untreated data for positions 10/90 and 63/90, versus the results from the Specialty Data (Fig. 1c–d). The Specialty and Treated results display good qualitative agreement, in that mutations at 90 (a primary drug-resistance mutation site) are positively selected regardless of whether an accessory mutation is present at 10 or 63, and each mutation reproducibly increases selection for the other mutation [25,31]. By contrast, in the Untreated dataset, mutations at codon 90 become unfavorable (negative selection pressure), while selection pressure for the accessory mutation (10 or 63) drops much less.

Although estimating conditional *K*_*a*_/*K*_*s *_accurately from the Treated dataset is difficult due to insufficient counts, we identified 274 codon pairs that showed statistically significant increases in conditional *K*_*a*_/*K*_*s *_for Treated relative to the Untreated dataset (Table 4). Of these shifts, eleven exhibited positive conditional selection pressure in Treated, but not in Untreated: 10→54, 71→54, 71→82, 63→90 (primary drug-resistance mutation codons); 20→10, 82→10, 71→10, 24→10, 10→63, 36→71, 63→73 (accessory drug-resistance mutation codons). And two codon pairs (93→63, an accessory drug-resistance mutation; 12→19, function unknown) are positively selected in both datasets. It is striking that almost all of these increases in conditional *K*_*a*_/*K*_*s *_involved known drug-resistance mutation sites.

**Table 4:** Significant differences of conditional selection pressure between Treated and Untreated dataset

Codon X	Codon Y	Treated			Untreated			p-value
			
		(*K*_*a*_/*K*_*s*_)_Y|Xa_	N_XaYa_	N_XaYs_	(*K*_*a*_/*K*_*s*_)_Y|Xa_	N_XaYa_	N_XaYs_	
20	10	5.21	74	7	0.08	1	6	0.000021
82	10	10.78	197	9	0.49	4	4	0.000512
71	10	4.39	285	32	0.42	23	27	<0.000001
24	10	25.12	51	0	0.25	2	4	0.000038
12	19	18.25	35	3	3.85	59	24	0.006913
10	54	7.81	164	9	0.19	4	9	<0.000001
71	54	3.65	162	19	0.14	4	12	<0.000001
10	63	12.42	353	14	2.15	70	16	0.000012
93	63	21.23	431	10	6.72	382	28	0.000995
36	71	24.13	196	4	0.46	14	15	<0.000001
63	73	2.76	112	20	0.10	4	19	<0.000001
71	82	10.73	196	9	0.59	6	5	0.000199
63	90	18.32	359	23	0.34	9	31	<0.000001

Due to the long list of significant shift results, only the 13 shifts that involved a positive conditional selection in Treated datasets are listed in this table.

### *K*_*a*_/*K*_*s *_comparison between the specialty and treated datasets

**Figure 2:**
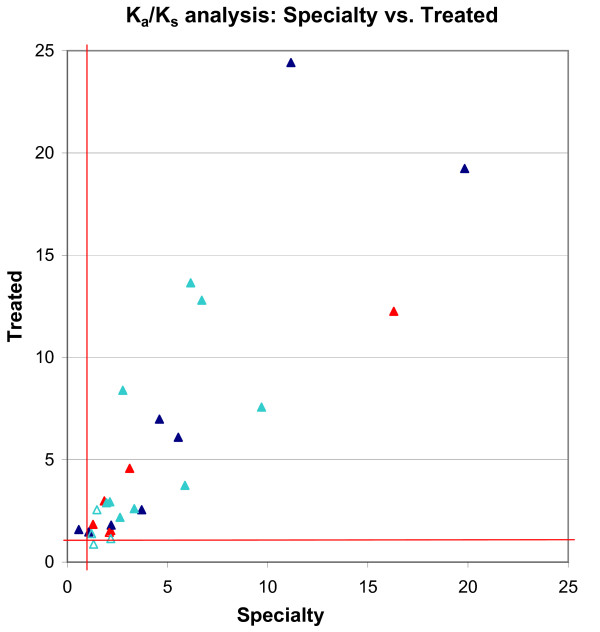
**Positive selection pressure in the Specialty andTreated datasets**. *K*_*a*_/*K*_*s *_values for positive selection pressures in the Specialty and Treated datasets are plotted. *K*_*a*_/*K*_*s *_values with a LOD < 2 in the Treated dataset were plotted as empty triangles. Different colors represent codons with different functions on drug resistance: Red: primary drug resistance mutation codon; Blue: accessory drug resistance mutation codon; Light blue: function unknown codon. The correlation coefficient of the selection pressures in these two datasets is 0.887, suggesting a significant quantitative agreement.

To evaluate the robustness of our approach, we compared its results from two independent datasets (Specialty; Treated), both from U.S. patients who received drug treatment. We first computed unconditional *K*_*a*_/*K*_*s *_values from the two datasets, and compared the results (Fig. 2). These independent data displayed a significant quantitative agreement, with a correlation coefficient of 0.887. Out of the 97 protease codons analyzed, only two significant differences were observed between the datasets. At codon 24, the Treated data showed mild positive selection (*K*_*a*_/*K*_*s *_= 1.59), but the Specialty dataset indicated slightly negative selection (*K*_*a*_/*K*_*s *_= 0.55). At codon 15, the situation was reversed (Treated *K*_*a*_/*K*_*s *_= 0.88; Specialty *K*_*a*_/*K*_*s *_= 1.30), but since both values were very close to neutral (*K*_*a*_/*K*_*s *_= 1.0), the difference was not statistically significant. The almost exact overlap in positive selection between the two datasets was highly significant (p-value = 10^-15.9^).

Overall, the positively selected *K*_*a*_/*K*_*s *_values computed from the Treated dataset tended to be slightly higher than those from the Specialty dataset. This is reflected in the ratios of *K*_*a*_/*K*_*s *_values from Treated vs. Specialty data, whose average (1.37) indicates that the Treated *K*_*a*_/*K*_*s *_values were 37% higher than the Specialty values. This is consistent with the fact that the Treated dataset consists exclusively of patients under drug treatment, whereas the Specialty dataset contains a mixture of samples from treated and untreated patients. Such mixing is expected to reduce the observed selection pressure arising from drug-treatment.

**Figure 3:**
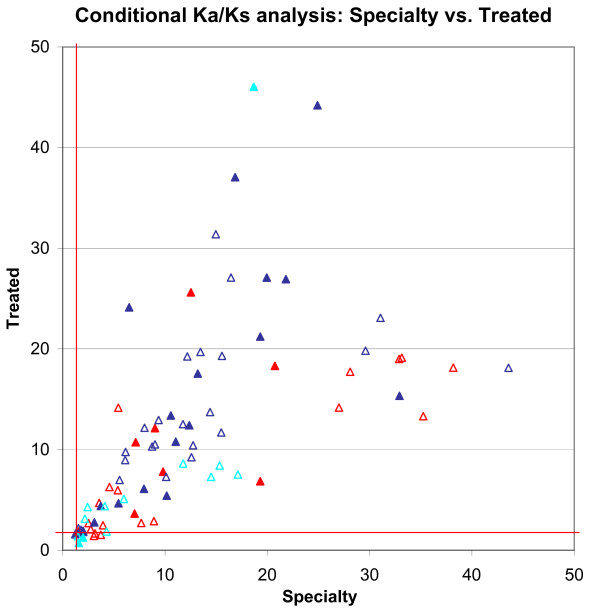
**Positive conditional selection pressure in the Specialty and Treated datasets**. (*K*_*a*_/*K*_*s*_)_Y|Xa _values for positive conditional selection pressures in the Specialty and Treated datasets are plotted. To filter the dataset to sites where (*K*_*a*_/*K*_*s*_)_Y|Xa _could be estimated with accuracy, only selection pressures with N_XaYa _+ N_XaYs _> 100 in both datasets were showed. An empty triangle indicated that we were not confident about the conditional selection value in the Treated dataset. The correlation coefficient of the conditional selection pressures in these two datasets is 0.495.

We next compared conditional *K*_*a*_/*K*_*s *_values from the two datasets (Fig. 3). These results display considerably more variation, but this appears to be due to insufficient counts in the Treated dataset for accurate estimate of conditional *K*_*a*_/*K*_*s *_values for many codon pairs. Whereas the unconditional *K*_*a*_/*K*_*s *_uses the whole dataset to estimate the rates of amino acid mutations (*K*_*a*_) and synonymous mutations (*K*_*s*_), the conditional *K*_*a*_/*K*_*s *_for a mutation *Y *conditioned on another mutation *X *is by definition limited to the small subset of sequences that contain mutation *X*. Since the Treated dataset contains only 1,797 samples, this drops many sites below the number of samples needed for accurate estimation of conditional *K*_*a*_/*K*_*s*_. To filter the dataset to codon pairs where (*K*_*a*_/*K*_*s*_)_Y|Xa _could be estimated with some accuracy, we restricted the analysis to cases where N_*YaXa *_+ N_*YsXa *_> 100. These results showed a strong match between conditional *K*_*a*_/*K*_*s *_values calculated from the two independent datasets, with a correlation coefficient of 0.495. None of the codon pairs with positive conditional selection in the Specialty dataset showed negative selection in the Treated dataset.

Again, the positively selected conditional *K*_*a*_/*K*_*s *_values computed from the Treated dataset were slightly higher than those from the Specialty dataset (by 17.9% on average). This is consistent with the fact that the Specialty dataset is a mixture of treated and untreated samples, whereas the Treated dataset was collected exclusively from treated patients.

### *K*_*a*_/*K*_*s *_between the untreated and Africa datasets

**Figure 4:**
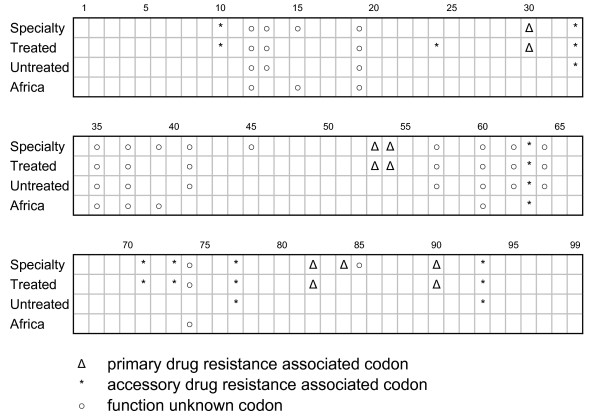
**Independent HIV-1 datasets yield reproducible positive selection results**. Our method identified positively selected sites (marked with different symbols depending on whether the site is a known drug resistance mutation, accessory mutation, etc.) in independent analyses of four different datasets: **Specialty**: 48,387 U.S. patient samples, a mix of treated and untreated patients; **Stanford-Treated**: 1797 U.S. patient samples with specific drug treatments; **Stanford-Untreated**: 2628 U.S. untreated patient samples; **African**: 399 African patient samples, HIV-1 subtype C (predominantly untreated patients).

We computed *K*_*a*_/*K*_*s *_values from two untreated datasets: 2,628 HIV-positive samples from U.S. patients who had received no drug treatment (Untreated); and 399 HIV-positive samples from African patients (predominantly untreated [18-20]). While the African dataset is too small for accurate estimation of *K*_*a*_/*K*_*s *_values, the two datasets show strong qualitative agreement (Fig. 4). Of the 97 protease codons analyzed, seventeen codons showed positive selection (*K*_*a*_/*K*_*s *_>1, LOD>2) in at least one of the two datasets. In twelve cases, both datasets indicated positive selection (*K*_*a*_/*K*_*s *_>1); in four cases, the Untreated data detected positive selection but the African dataset did not; and in one case (codon 39), the reverse was observed. This high level of agreement was statistically significant (P-value = 10^-3.75^). Despite the fact that these data were collected on different continents and represent different HIV-1 subtypes (B in Untreated, and C in African), they indicate that fitness mutations are found mostly at the same positions in the two populations.

### Assessing the effects of mixing treated and untreated data on selection pressure calculations

**Figure 5:**
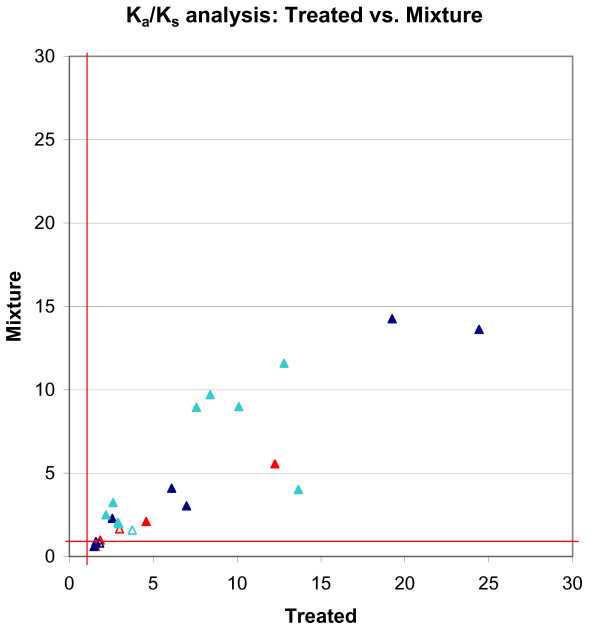
**Effects of mixing Treated and Untreated data on selection pressure calculation**. We have mixed the Treated (1797 samples) with the Untreated (2628 samples) to assess the effects of a mixed data on selection pressure analysis. This mixing caused up to two-fold decreases in positively selected Ka/Ks values (compared with the Treated dataset), with shifts averaging of 32.7%. Out of the 24 positively selected codons in the Treated dataset, only two became negatively selected in the Mixture dataset. *K*_*a*_/*K*_*s *_values with a LOD < 2 in the Mixture dataset were plotted as empty triangles.

In principle, the *K*_*a*_/*K*_*s *_analysis approach differs from associational studies in that it does not require an untreated "reference" dataset in order to detect drug-resistance signals, and should even be tolerant of significant levels of contamination by untreated samples. To test its robustness to such contamination, we deliberately mixed the Treated and Untreated datasets, and analyzed *K*_*a*_/*K*_*s *_from the mixed data. We tested several mixtures: adding 25% contamination of Untreated samples (i.e. *N*_*untreated *_= 0.25 *N*_*treated*_); 50% contamination; and 146% contamination (i.e. all the available Untreated samples). The 25% and 50% mixtures caused only minor effects on the measured Ka/Ks values (data not shown). The maximum contamination (146%) caused up to two-fold decreases in positively selected *K*_*a*_/*K*_*s *_values (Fig. 5), with shifts averaging of 32.5%. Out of the 24 codons detected to be positively selected in the Treated dataset, only two became negatively selected in the mixture dataset. These data show that the *K*_*a*_/*K*_*s *_approach is relatively robust even to very high levels of contamination by untreated samples.

## Discussion

Naïve *K*_*a*_/*K*_*s *_analysis involves several assumptions that can undercut the robustness of its results. In particular, the assumption of a single consensus sequence as the common ancestor of all sequences in the data sample ignores the possibility of important phylogenetic relationships among these sequences. Phylogenetic structure in the dataset thus can cause artifacts in *K*_*a*_/*K*_*s *_analysis, and some methods perform *K*_*a*_/*K*_*s *_analysis in the context of an explicit phylogeny [33]. Linkage disequilibrium between sites can also cause artifacts in conditional *K*_*a*_/*K*_*s *_analysis, by creating correlations between sites that are purely historical, and not due to functional interaction [34,35]. These problems are likely to be most severe in genomes with a low mutation rate and low recombination rate, and less severe in genomes with very high mutation and recombination rates, such as HIV.

To assess these problems, we have used HIV mutation data to test the utility and robustness of conditional *K*_*a*_/*K*_*s *_analysis, by comparison with known functional interactions involved in drug resistance, and comparison of the *K*_*a*_/*K*_*s *_results for multiple independent datasets. Several lines of evidence indicate that, at least for HIV protease, conditional *K*_*a*_/*K*_*s *_analysis provides robust detection of real functional interactions that are not artifacts: **1**) *K*_*a*_/*K*_*s *_identification of sites with strong positive selection correctly identified known drug resistance mutations in HIV protease (p-value = 10^-3.3^; [10]); **2**) *K*_*a*_/*K*_*s *_identification of mutations with strong positive selection matched independent experimental phenotypic assays [10,36]; **3**) conditional *K*_*a*_/*K*_*s *_identification of pairs of sites with strongly positive conditional selection ratios ((*K*_*a*_/*K*_*s*_)_Y|X _>>1) strongly matched independent experimental studies identifying pairs of mutations that are correlated in treated vs. untreated patient datasets [9]; **4**) detailed functional predictions from conditional *K*_*a*_/*K*_*s *_(such as distinguishing primary vs. accessory drug resistance mutations) matched well-known examples in HIV protease where such detailed functional information is available; **5**) conditional *K*_*a*_/*K*_*s *_values computed from independent datasets (Specialty vs. Stanford-Treated) yielded highly similar results, indicating that these values are not artifacts, but broadly reproducible characteristics of HIV evolution in the wild population; **6**) the strong positive selection patterns identified by conditional *K*_*a*_/*K*_*s *_in treated patients (Specialty; Stanford-Treated) vanished in the untreated patient dataset (Stanford-Untreated), indicating that these *K*_*a*_/*K*_*s *_patterns genuinely reflect selection pressure associated with drug treatment. Considered as a whole, all of these assessments suggest that conditional *K*_*a*_/*K*_*s *_robustly identifies genuine functional selection pressure in HIV protease, not artifacts, and thus may also be useful in other genes with similar characteristics, such as other HIV genes, or other rapidly-evolving viruses. However, it is likely that a more sophisticated approach, incorporating phylogenetic structure, will be required for other types of problems.

The high level of reproducibility of *K*_*a*_/*K*_*s *_results across four independent datasets in this study (Fig. 4) merits further consideration. These datasets were collected entirely independently, and come from diverse sources [9,10,18-20]. The quantitative agreement between *K*_*a*_/*K*_*s *_values obtained from these datasets is surprisingly consistent considering that the input data were collected by different investigators, from different patient groups, over different time periods, under differing drug treatment regimens (and in the case of the Specialty dataset, no treatment information available whatsoever), and even from different continents and HIV-1 subtypes. Even the weakest level of agreement between datasets (Untreated vs. Africa, by far the smallest dataset) was significant (p-value = 10^-3.75^). Moreover, comparison of the *K*_*a*_/*K*_*s *_results versus known drug-resistance mutations demonstrates that this approach predicts true drug-resistance mutation codons with a high level of accuracy (13 drug-resistance mutation codons in *K*_*a*_/*K*_*s *_analysis, and 19 in the conditional *K*_*a*_/*K*_*s *_analysis). These results also show that comparison of *K*_*a*_/*K*_*s *_values between treated and untreated datasets is effective at distinguishing primary and accessory drug-resistance mutations (only observed in the treated dataset) from viral fitness mutations (observed in the untreated dataset). This suggests that this approach could be applied successfully to more specific dataset comparisons (such as two different drug treatments).

These four datasets also suggest that viral fitness mutations in HIV protease are surprisingly consistent throughout HIV-1 populations. In general, mutations that were positively selected in untreated samples (i.e. the Untreated and African datasets) were also observed to be positively selected in treated samples (i.e. in both the Specialty and Treated datasets). To a first approximation, these mutations seem to represent a uniform background of positive selection pressures that are consistently observed in any set of samples we have analyzed. Furthermore, these "viral fitness" mutation codons matched strongly between the Africa and Untreated datasets, indicating that these fitness pressures are even consistent across different HIV-1 subtypes (B in Untreated vs. C in Africa) [37,38].

These comparisons suggest that it may be useful to classify mutations according to their selection pressure values in treated vs. untreated patient populations. For example, whereas mutations that are positively selected even in untreated samples may be classed as improving viral fitness [37], mutations that are positively selected in treated samples but not untreated samples are likely to be drug-resistance mutations [13-15]. It is also interesting to classify mutations according to whether their positive selection effects are conditional or unconditional. A mutation that is positively selected by drug treatment, with no conditional dependence on any other mutation, evidently makes a primary (i.e. direct) contribution to drug-resistance [13-15]. By contrast, a mutation that is positively selected by drug treatment only in the presence of a primary mutation, evidently does not make a direct contribution to drug-resistance, but rather a secondary (or "accessory") contribution mediated by the primary mutation [16,17]. Finally, a mutation that compensates for a deleterious fitness effect caused by a primary drug-resistance mutation can also be distinguished by *K*_*a*_/*K*_*s *_analysis [31]. Such a mutation should not exhibit unconditional positive selection in either untreated or treated samples, but should show positive *conditional *selection (only in the presence of the primary mutation) in both types of samples.

How much "mixing" of sample types can this approach tolerate? We have tested the effects of deliberately mixing treated and untreated datasets upon positive selection mapping by *K*_*a*_/*K*_*s*_. These data show that *K*_*a*_/*K*_*s *_can robustly detect drug resistance mutations even when the level of mixing equals or even exceeds 100%. Such mixing reduced *K*_*a*_/*K*_*s *_estimates somewhat, but qualitatively the results were robust. Thus, not only can *K*_*a*_/*K*_*s *_detect drug resistance mutations in a single dataset (with no requirement for a reference dataset of untreated samples), it can do so even when the primary dataset is contaminated with a large fraction of untreated samples, or samples with different treatments. This can be very useful in situations where treatment information is incomplete or unavailable.

Since different amino acid mutations can have different functional effects [36], an obvious extension of this analysis to consider the conditional selection pressure effects of individual amino acid mutations, rather than treating all amino acid mutations at one site as equivalent, as was done in this analysis. Such an extension would yield detailed information about the amino acid interactions between two codon sites [10].

Conditional Ka/Ks represents a slight extension of existing work on co-evolution of amino acid residues in protein sequences. One well-established approach to this problem is the use of correlation analysis to look for correlations between amino acid residues in a set of related sequences [17,39-44]. For example, by analyzing mutation correlation in an alignment of 266 non-redundant SH3 domain sequences, Larson et al. were able to predict residue-residue contacts in the structure of the SH3 domain [40]. Such correlated mutation analysis tests for non-additive effects of a pair of mutations on reproductive fitness, such as epistasis [45] or compensation [43]. One important question not addressed by correlation analysis is whether an interaction between a pair of mutations is symmetric or asymmetric; that is, whether the influence of one mutation on the fitness effect of the second mutation is the same as the influence of the second mutation upon the fitness effect of the first. Since the definition of correlation is inherently symmetric (i.e. the correlation coefficient ρ(*x,y*) = ρ(*y,x*) is invariant to exchange of *x *and *y*), it does not answer this question. Conditional Ka/Ks combines the benefits of correlation analysis (detection of interactions between sites) with the benefits of standard Ka/Ks (a measurement of functional selection pressure). Moreover, this method can distinguish symmetric vs. asymmetric functional interactions.

We emphasize that in the simple form presented in this paper, conditional Ka/Ks is probably not applicable in datasets with a low mutation rate or strong linkage disequilibrium. If the number of mutation observations at a site is small, results will not be statistically significant. Worse, if individual observations of a mutation are not independent mutation events, but instead arose by common descent from a single ancestral mutation event, the results will be skewed by such "double-counting" bias within the data. In sequence datasets with clear phylogenetic structure, it will be essential to compute conditional Ka/Ks for specific branches of the phylogenetic tree, as has been done for standard Ka/Ks calculations [33,46]. This improved approach control for departures from conditional independence in the data sample, by explicitly analyzing the conditional dependence structure (i.e. the phylogenetic tree). Thus conditional Ka/Ks need not be limited to cases that fit a "star topology", but can be generalized to work with phylogenetic trees.

## Conclusion

Conditional *K*_*a*_/*K*_*s *_analysis can detect mutation interactions between different sites. The analysis results also reveal important effects on fitness that are asymmetric and are very useful to predict primary vs. accessory drug resistant mutations. Comparison of *K*_*a*_/*K*_*s *_values between treated and untreated datasets is effective at distinguishing drug-resistance associated mutations from viral fitness mutations. Analysis results from four completely independent datasets are highly consistent both qualitatively and quantitatively. These results can be very helpful to understand the functional roles of different mutations in the development of drug resistance.

## Authors' contributions

L. Chen carried out the sequence alignment, mutation detection, selection pressure and conditional selection pressure analysis. C. Lee proposed the conditional Ka/Ks metric, participated in the design of the study, and drafted the manuscript.

## Reviewers' comments

### Reviewer's report 1

Wen-Hsiung Li (nominated by Eugene V. Koonin), University of Chicago, IL, USA  

Second review following revisions:  

This is a very interesting study. The author developed a conditional Ka/Ks test and applied it to large amounts of HIV sequence data to detect drug resistance mutation and fitness mutations. Since the MS reads well and since I am traveling, I have not read it carefully, but I suppose there are no serious problems.

My main suggestion is that the authors should explain in more detail the methodological parts, so that readers can understand them better.

Minor comments:

Give an explanation of Ka and Ks in the Introduction.

Explain the definition of q on page 7.

In many places "amino acid mutations" should be "amino acid substitutions or replacements".

#### Author response

*We have briefly introduced Ka/Ks in the Background and there is detailed description of how to calculate Ka/Ks in the Method. We have defined the mathematical formula to calculate q on page 7*.

### Reviewer's report 2

Robert Shafer (nominated by Eugene V. Koonin), Stanford University, CA, USA  

Second review following revision

The manuscript has been greatly improved by addressing the larger specific issues raised in the initial review and also by improving the presentation of the results and clarity of the writing. Below I've summarized some of the specific improvements and do raise two additional points – the second of these points should definitely be addressed because it will be raised by others knowledgeable about the specific patterns of mutations observed in drug-resistant isolates.

1. Phylogenetic relationships among the sequences. The manuscript has been improved by discussing the relevance of this issue and by adopting a heuristic approach – excluding samples with >98% sequence identity. The authors should add to the methods that this step was taken.

2. Inherent variability of HIV-1 sequences. The authors have explained in more detail how conditional Ka/Ks was determined. Although their approach may remain controversial, it is certainly novel and worthy of publication and broader discussion.

#### Author response

*As suggested by the reviewer, we have added a sentence in the Method Section to explain that we have excluded samples with >98% sequence identity*.

3. Availability of the data. Although this may be partly beyond their control, the authors' attempts to make the data available – ideally the complete nucleotide sequence – is laudatory.

#### Author response

*All the sequence data and analysis results will be available to open access upon the publication of this manuscript at: *

4. Presentation of the results. This has been improved and the Additional File is also useful.

Several additional comments:

The authors state in the methods that "we only counted single nucleotide substations". Would this influence their ability to detect positions at which the mutations are 2-bp mutations. If so this should be stated. This would not influence the protease because, as far as I am aware, the 2-bp changes I47A, I54A, V82S, I84A/C are each uncommon mutations at positions at which 1-bp changes also cause resistance (I47V, I54V, V82A, I84V). But it would influence a similar analysis on RT as T215Y and T215F are each 2-bp changes and are the only drug-resistance mutations at these positions.

I believe the authors need to add further explanation as to the implications of conditional Ka/Ks because of the following: In table 1, several of the Codon X's are actually known to follow the Codon Y. Therefore the statistical dependence of Y on X, does not necessarily mean that Y follows X. The most obvious example of this is the relationship between position 30 and 88. There is only one mutation at position 30 (D30N). There are several at position 88 (N88D >> N88S >>> N88T/G). N88S occurs completely independent of D30N but is much less common than N88D. However, N88D always follows and never precedes D30N. Therefore there are two paths to resistance involving these mutations: D30N followed by D30N+N88D and N88S alone. I believe that this is also the case for codons 73 and 90 (73 nearly always follows 90) and 24 and 82 (24 nearly always follows 82).

#### Author response

Only a very small fraction of the mutations involve multiple nucleotide substitutions in a codon (about 10%). They are excluded from this analysis because their evolution patterns are different from single nucleotide polymorphism (ex. It takes two steps for ctc mutating to gcc, which could be either of these two processes: 1. ctc→gtc→gcc; 2. tct→ccc→gcc)

### Reviewer's report 3

Shamil Sunyaev, Harvard Medical School, MA, USA  

Second review following revisions:

This manuscript presents an analysis of HIV protease sequencing datasets. Comparing synonymous and non-synonymous sequence changes, the authors detected sites under putative positive selection possibly in response to drug treatment.

The manuscript is based on the earlier work by the same authors (J Virol. 2004 Apr;78(7):3722–32.) and introduces a new concept of conditional positive selection. The authors argue that selection in some sites is conditional on amino acid residue at a different site.

This is an interesting work and the manuscript has improved upon revision. The revised manuscript includes discussion of the existing literature on co-evolution of amino acid residues. However, I would still like to make a few comments, some of them being critical.

1) A few comments on the method

a) The authors make a series of simplifications including assumptions of star-like phylogeny and absence of multiple substitutions in individual codons. Therefore, the method assumes that all sequences are descendants of a single sequence and the estimation of Ka and Ks is done by simple counting. I agree with the authors that currently available methods are not applicable to the dataset of ~50,000 sequences. Thus, the authors have no other choice rather than to rely on simpler computationally efficient methods and making these assumptions is a necessity. The authors also point that the number of codons with multiple substitutions is not expected to be high. However, if the above assumptions are not valid (and it is not clear why the phylogeny is expected to be star-like), the results of the analysis have to be received with caution. Inference of positive selection can be erroneous. Analysis of statistical significance which relies on the binomial distribution and application of the Fisher exact test are not justified if these assumptions are not satisfied.

#### Author response

*We have identified all mutations (both single nucleotide polymorphisms and multiple nucleotide polymorphisms) in this dataset. The results show that only a very small fraction of the mutations involve multiple substitutions in a codon (about 10%). They are excluded from this analysis because their evolution patterns are different from single nucleotide polymorphism*.

*HIV has extremely high mutation rate and high recombination rate. And it is under strong selection pressure, especially when the patient is under drug treatment. These same factors tend to make HIV evolution within a specific subtype follow the star topology model*.

b) It is my understanding that frequencies of transition and transversion rates were estimated using both synonymous and non-synonymous changes. It may be better to use synonymous changes only.

#### Author response

*As suggested by the reviewer, we have estimated the transition and transversion rates only using synonymous changes (F*_*t*_/*F*_*v*_*= 23.75). This does not alter the results of selection pressure and conditional selection pressure analysis much. For the purposes of this paper (selection pressure analysis) we prefer to use the more conservative assumption of measuring F*_*t*_/*F*_*v*_*from all nucleotides (F*_*t*_/*F*_*v *_= *8.90), since this matches what has been published previously (Wilson, J. W., J Clin Microbiol, 2000)*.

c) Testing all pairs of codons creates an obvious multiple test problem. The manuscript would probably benefit from the discussion on how the rate of false-positive predictions is controlled given this multiple test problem.

2) It is of interest whether amino acid residue pairs which display conditional selection located close to each other in protein structure. The structural analysis can serve as a confirmation of the conclusions made by the authors. However, I agree with the authors's response that this should be a subject of a separate study.

3) Most importantly, I do not find the explanation of the observed "conditional selection" satisfactory. The authors argue that mutations with significant "conditional Ka/Ks values" are not directly involved in the drug resistance and play accessory role. The first mutation which is directly involved in the drug resistance is destabilizing (in other words, is expected to be deleterious in the absence of the drug treatment) and the second mutation is needed to compensate the destabilizing effect of the first mutation. Although, this is certainly a possibility, it is feasible to imagine many other scenarios. For example, is it possible that the first mutation enables the effect of the second mutation on the drug response, i.e. the first mutation is not destabilizing but the second mutation would not have an effect on drug resistance without the first mutation? Alternatively, is it possible that the first mutation is not destabilizing, whereas the second mutation would be destabilizing (and deleterious) in the absence of the first mutation (Dobzhansky-Muller incompatibility)? In this case also the second mutation may be directly involved in drug resistance. Importantly, the latter scenarios do not involve initial increase in frequency of a destabilizing variant.
